# MiR-137 promotes anoikis through modulating the AKT signaling pathways in Pancreatic Cancer

**DOI:** 10.7150/jca.44037

**Published:** 2020-08-28

**Authors:** Lin Li, Zhiwei He, Changhao Zhu, Shiyu Chen, Zhehao Yang, Jing Xu, Ningrui Bi, Chao Yu, Chengyi Sun

**Affiliations:** 1 Guizhou Medical University, Guiyang, China.; 2 Department of Hepatic-Biliary-Pancreatic Surgery, The Affiliated Hospital of Guizhou Medical University, Guiyang, China.; 3 Key Laboratory of Hepatobiliary and Pancreatic Surgery, Guiyang, China.; 4 College of Basic Medicine, Guizhou Medical University, Guiyang, China.

**Keywords:** microRNA-137, paxillin, pancreatic cancer, anoikis

## Abstract

Anoikis resistance is a fundamental feature of the survival of metastatic cancer cells during cancer progression. However, the mechanisms underlying anoikis resistance in pancreatic cancer (PC) are still unclear. MicroRNA-137 (miR-137) is a tumor suppressor that inhibits the proliferation and invasion of cancer cells through targeting multiple oncogenes. However, the effects and molecular mechanism of miR-137 on anoikis of PC are still unclear. Here we demonstrated that miR-137 was downregulated after the induction of anoikis model in time dependent. Function assays revealed that miR-137 promoted the pancreatic cancer cells anoikis *in vitro* and vivo. According to bioinformation analysis of clinical databases, we predicted that paxillin (PXN) was a target of miR-137. Further, TCGA analysis revealed that PXN was closely associated with the development of PC. Through loss-of-function studies, we demonstrated that PXN was a functional target of miR-137 on anoikis of PC cells. Moreover, we found that PXN promoted the activation of the AKT signaling pathways which was involving in the cancer cells anoikis. Together, our findings reveal that miR-137 plays a novel role during anoikis and may serve as a potential target for the detection and treatment of PC.

## Introduction

PC is highly invasive malignancies, and the dismal 5-year overall survival rate is 7%. The lack of early diagnostic symptoms and early metastatic behavior often prevent curative resection, and approximately 50% of patients with PC have metastases at diagnosis 1, 2. Metastasis is a complex multi-step process that requires the regulation of tumor cell migration, adhesion, invasion, and evasion of the immune system 3. During metastasis, cancer cells pass through the primary tumor, infiltrate, metastasize, extravasate, settle, and proliferate in the circulatory system 4. Therefore, understanding the molecular mechanisms of the key steps in PC metastasis will help improve the prevention of PC and guide efforts to develop effective treatments.

Anoikis refers to cell death caused by the inability of cells to adhere to the extracellular matrix 5. Therefore, anoikis is a key cellular program that can ensure the physiological integrity of tissues by eliminating cells dissociated from their original location 6. Notably, anoikis resistance is vital step during cancer progression and metastatic colonization 7. Moreover, anoikis is an important barrier to the formation of distant metastasis 8.

MicroRNAs (miRNAs) are small (approximately 19-25 nucleotides), endogenous noncoding RNAs 9. Generally, miRNAs are abnormally expressed in many diseases such as multiple cancers 10. Considerable evidence indicates that miRNAs act as tumor suppressors or oncogenes. Research from Cui S et al. suggested that miR-137 had a suppressive role in liver cancer via targeting EZH2 11. Results demonstrated that mir122, mir137, and mir206 contributed to tissue characteristics and carcinogenesis by regulating pyruvate kinase M1/2 (PKM) expression 12. While miRNAs can inhibit gene expression by directly inducing the degradation of mRNA or by base-pairing with complementary sites in the 3′-untranslated region (3′-UTR) of the target mRNA 13. Strikingly, we confirmed miR-137 targeted the 3′-UTR of the PXN mRNA. PXN is a multifunctional and multidomain focal adhesion adapter protein which serves as an important scaffolding role at focal adhesions by recruiting structural and signaling molecules involved in cell movement and migration, when phosphorylated on specific Tyr and Ser residues 14. Wei W et al. reported that WNT5A/JNK signaling initiated cell migration of PC through activation of PXN 15. However, to date, few studies have explored the role of miR-137 in anoikis. In the previous study, the levels of miR-137 were significantly lower in PC tissue and associated with the inhibition of the proliferation of PC as well as its invasiveness, ability to metastasize, and resistance to anticancer drugs 16. The resistance of cancer cells to anoikis allows them to invade tissues and subsequently metastasize 17.

In the present research, we report that miR-137 promotes anoikis of PC cells *in vitro* and vivo, and regulates the AKT signaling pathways in PC. Further, there is an increasing evidence that miRNAs may serve as effective biomarkers for diagnosing cancer, targets for cancer treatment, or both 18, 19.

## Materials and Methods

### Cell culture and clinical specimens

The PANC-1 and AsPC-1 cell lines derived from PC were acquired from the Department of Biliary and Pancreatic Surgery, Tongji Hospital, Huazhong University of Science and Technology. PANC-1 cells were maintained in DMEM (Gibco, New York, USA) supplemented with 10% fetal bovine serum (Gibco, New York, USA). AsPC-1 cells were cultured in RPMI 1640 medium (Gibco, New York, USA) supplemented with 10% fetal bovine serum 37 °C in an atmosphere containing 5% CO2. Twenty-three sets of clinical specimens (cancer tissue and corresponding para-cancerous tissue) from PC patients who have not received chemotherapy or radiotherapy were collected from the Affiliated Hospital of Guizhou Medical University and immediately stored in liquid nitrogen until analysis.

### Cell transfection

Cells (2×10^5^) were seeded in 6-well plates on the day before transfection with siRNAs (Ribobio, Guangzhou, China). For luciferase reporter assays, pairs of oligonucleotides containing the 3′-UTR binding site for miR-137 (Ribobio, Guangzhou, China) were used. Transfection of siRNAs was performed using lipofectamine 3000 (Life Technologies Co., Carlsbad, USA) according to the manufacturer's instructions. The siRNAs were transfected 48 h. The sequence of miR-137 NC mimic: 5′-UCACAACCUCCUAGAAAGAGUAGA-3′, the sequence of miR-137-mimic: 5′-UUAUUGCUUAAGAAUACGCGUAG-3′, the sequence of miR-137 inhibitor: 5′-CTACGCGTATTCTTAAGCAATAA-3′, the sequence of small-interfering RNA sequence of PXN: 5'-CATACCCAACTGGAAACCACACATA-3', and the sequence of PXN control: 5'-AACGTACGCGGAATACTTCGA-3'. MiR-137 upregulated lentiviruses (miR-137), negative-control lentiviruses (NC) and were purchased from Genechem (Shanghai, China). All transfections were performed according to the manufacturer's instructions.

### RNA isolation, reverse transcription, and qPCR

The PC cell lines PANC-1 and AsPC-1 were divided into an overexpression group (miR-137) and a negative control group (NC). Exponentially growing cells (2×10^5^) were used to seed a six-well plate. After overnight culture, 1μl viral constructs (Shanghai Genechem Co., Ltd.) were put into cell medium according to the manufacturer's instructions. Total RNA was extracted using TRIzol (Invitrogen, Carlsbad, CA, USA) reagent according to the manufacturer's instructions. Reverse transcription of microRNAs was performed using PrimeScript RT Master Mix (Takara, Japan). MiR-137 overexpression constructs, a 361-bp fragment up and downstream of the pre-miR-137 was amplified from PC cells complementary DNA (cDNA) by PCR (forward primer, 5′-GCTCAGCGAGCAGCAAGAGT-3′ and reverse primer, 5′-GGCAATAAGAGCGAAACACCA-3′). QPCR was conducted using SYBR Green (Takara, Japan) according to the manufacturer's instructions. We constructed PXN siRNAs (Ribobio, Guangzhou, China) to transfect PANC-1 and AsPC-1 cells, which were designated the si-PXN group and the control group, respectively. Exponentially proliferating cells (2×10^5^) were used to seed a six-well plate. After overnight incubation, the PXN siRNAs were used to transfect cells according to the manufacturer's instructions. The primer sequences were as follows: si-PXN: 5′-CTGCTGGAACTGAACGCTGTA-3′ (forward), 5′-GGGGCTGTTAGTCTCTGGGA-3′ (reverse); control 5′-GGAGCGAGATCCCTCCAAAAT-3′ (forward), 5′-GGCTGTTGTCATACTTCTCATGG-3′ (reverse). QPCR assays were conducted in the same manner described above.

### Western blotting

Total cell proteins were extracted at 4 °C using RIPA lysis buffer (Solabio, Beijing, China) containing protease and phosphatase inhibitors (Roche, Basel, Switzerland). Proteins were resolved using 8%-12% SDS polyacrylamide electrophoresis and electro-transferred to polyvinylidene difluoride (PVDF) membrane (Millipore, Bedford, MA). Then we blocked the blots with 5% non-fat milk for 1 h at room temperature. Western blots were probed with antibodies against p-AKT (Ser473, 1:1000, CST, USA) and total AKT (1:1000, CST, USA), both from Cell Signaling Technology), GAPDH (1:1000, CST, USA) at 4°C overnight. After washing, the blots were then incubated with the secondary antibody, goat anti-mouse (1:2000) and goat anti-rabbit (1:2000) (Boster Biological Technology co., ltd, USA) for 2 h at room temperature. Western blotting was performed at least three biological replicates.

### *In vitro* model of anoikis

Anoikis was induced using poly-HEMA (30 mg/mL, 2 mL in 95% ethanol; Sigma-Aldrich). Poly-HEMA was evenly spread on a six-well plate placed on an ultraclean workbench. After the alcohol was completely evaporated, the above operation was repeated until the poly-HEMA completely coated the bottom of the culture plate. The plates were sealed and stored at 4 °C. The wells were washed three times with PBS and UV-sterilized for 1 h. The digested cells (2 × 10^5^) were counted, and the assays were performed 24 h to 48 h later.

### Flow cytometry

Floating cells were harvested by centrifugation at 300 × g, 18 °C for 5 min. Attached cells were trypsinized, and then centrifuged at 300 × g, 18 °C for 5 min. And 1 × 105 cells were resuspended in 500 μl of 1× Binding Buffer, 5μl of Annexin V-FITC (Hangzhou Lianke Biotechnology Co., Ltd) and 10μl of PI (Hangzhou Lianke Biotechnology Co., Ltd.), and then incubated at room temperature for 5 min in the dark, following the manufacturer's instructions. The cells were analyzed using flow cytometry (Cytomics FC 500, CA, USA).

### Suspension culture assays

Cells (500 per well) were seeded into 6-well, ultralow attachment cluster plates (Corning, NY, USA) and cultured in complete DMEM medium (Invitrogen, Carlsbad, CA, USA). After one week, cells were photographed and counted.

### *In vivo* model of anoikis

Four-week-old BALB/c female nude mice (Beijing HFK Bioscience Co., Ltd) were divided into two groups, ten per group. PANC-1 cells were divided into NC and miR-137 groups, 1 × 106 of each were intraperitoneally injected, and the mice were observed each week and weighed. After 8 weeks, the surviving mice were killed using cervical dislocation. All mice were dissected and checked for tumor formation on the abdominal cavity.

### Immunohistochemistry

Paraffin-embedded formalin-fixed tissue sections were dehydrated, subjected to antigen retrieval, blocked with 3% H_2_O_2_, washed with serum, and incubated with primary-antibody working solution at 4 °C overnight. The slides were rewarmed to room temperature, incubated with secondary antibody at 37 °C for 1 h, and washed twice with PBS for 5 min. One drop of DAB Plus Chromogen was added to 1 mL of DAB Plus Substrate, and the mixture was added to each slide for 3 min to 15 min. The slides were washed with tap water, counterstained, differentiated, stained nuclear with hematoxylin, dehydrated, and covered with a cover slip.

### Tunel assays

All tumors were embedded in paraffin. After slides were produced, they were stained with hematoxylin and eosin (H&E). The assays were performed with the In-Situ Cell Death Detection Kit, Fluorescein (Roche Molecular Biochemicals, Penzberg, Germany) using the manufacturer's instructions. The slides were counterstained with propidium iodide (Vector Laboratories Inc., Burlingame, CA, USA). The slides were evaluated with confocal microscopy.

### Luciferase assays

A pGL3-3′UTR-PXN luciferase gene reporter vector was constructed, and the corresponding predicted miR-137 binding site was mutated to generate the pGL3-3′UTR-PXN mut luciferase gene reporter vector. The miR-137 expression vector pcDNA3.0-miR137 was used to transfect cells along with pLa3-3′UTR-PXN or pGL3-3′UTR-PXN mut. Cells transfected with the reporter construct that expressed renilla reniformis fluorescent protein served as a control.

### Statistical analysis

Differences in the three or four groups were tested using ANOVA and the Student *t* test was performed to analyze the significance of differences in the two groups between the mean values of three independent experiments. The correlation between PXN and miR-137 were analyzed by person correlation coefficient. A significant difference was defined as *P<*0.05. All analyses were performed using Graphpad Prism (version 7.00). Survival estimates were calculated using two-stage test.

## Results

### Overexpression of miR-137 promotes anoikis of PC cells *in vitro*

**Figure 1:**
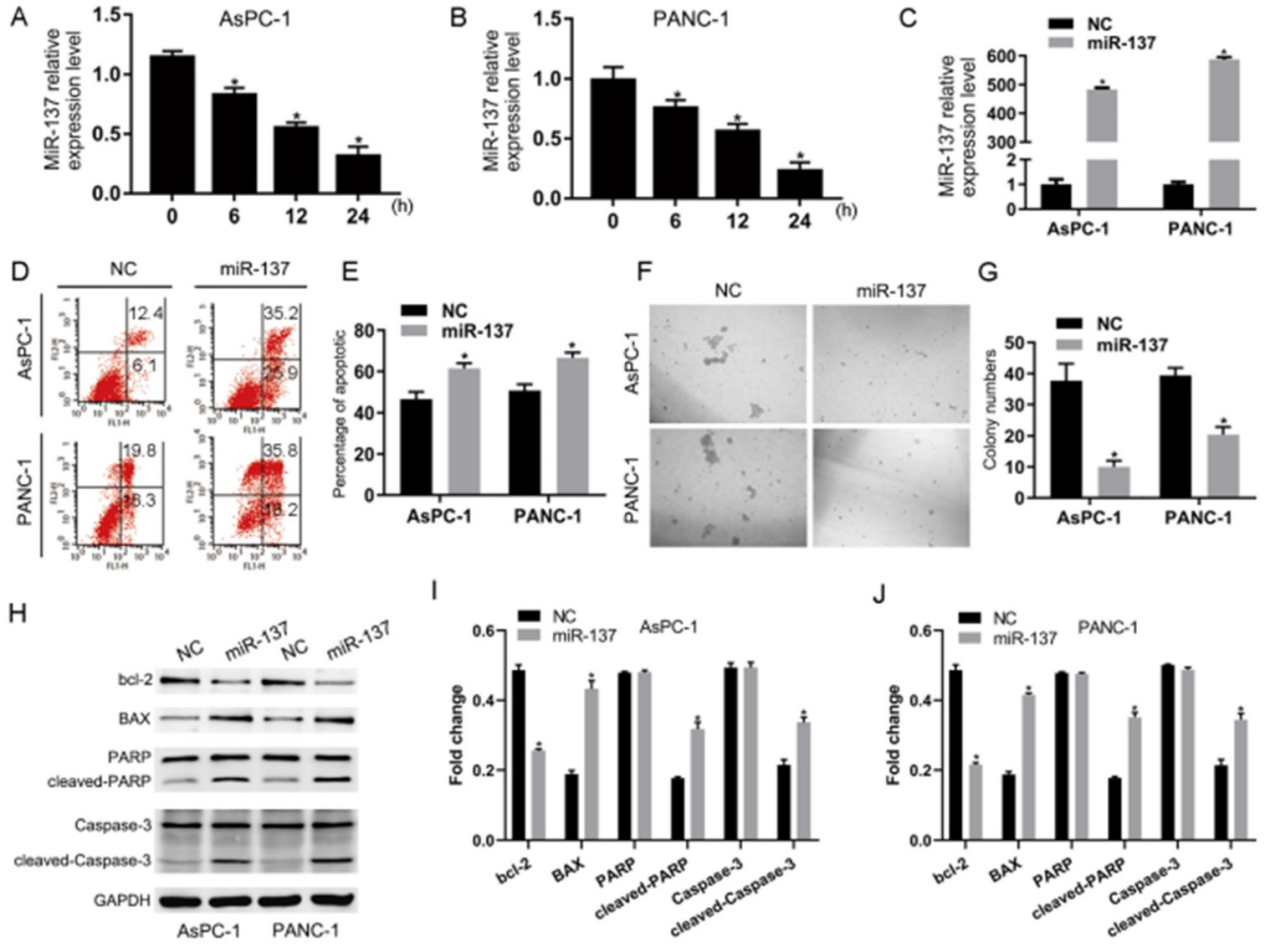
** Overexpression of miR-137 promotes anoikis of PC cells *in vitro*. A-B.** Q-PCR analysis of the levels of miR-137 expression in the AsPC-1 and PANC-1 cells induced anoikis. **C.** Q-PCR analysis of the relative levels of miR-137 expression in the AsPC-1 and PANC-1 cells induced anoikis after transfected by lentiviral. **D-E.** Annexin V-FITC/PI staining of the AsPC-1 and PANC-1 cells after the anoikis assays. Error bars represent the mean ± S.D. of three independent experiments. The apoptotic cells percentage was assessed by flow cytometric analysis. The percentage of early apoptotic cells (Annexin V+/PI-, LR) and late apoptotic plus dead cells (Annexin V+/PI+, UR) were shown in the representative flow cytometry figure. **F-G.** Cellular morphologies of AsPC-1 and PANC-1 cells induced anoikis in suspension conditions captured by microphotography. **H-J.** Western blotting analysis the expression of BCL-2, BAX, PARP, cleaved PARP, Caspase-3 and cleaved Caspase-3 in the AsPC-1 and PANC-1 cells induced anoikis. GAPDH served as the loading control. *P<0.05. All data were expressed as the mean±SD of three experiments, and each experiment included triplicate repeats.

To investigate the potential roles of miR-137 in the anoikis of PC, we first examined the expression levels of miR-137 in AsPC-1 and PANC-1 cells which had been induced anoikis. The qPCR results showed that the expression levels of miR-137 were reduced (Fig. 1A-B). To futher explore the impact of miR-137 on anoikis of PC cells, we stably expressed miR-137 in AsPC-1 and PANC-1 cells by lentiviral infection (Fig. 1C). Flow cytometry assays demonstrated that overexpression of miR-137 increased anoikis apoptosis cells (Fig. 1D-E). Correspondingly, suspension culture assays revealed that overexpression of miR-137 decreased cells formation of anoikis of AsPC-1 and PANC-1 cells (Fig. 1F-G). Consistently, apoptotic protein including bcl-2 associated x protein (BAX), cleaved-poly ADP-ribose polymerase (cleaved-PARP), cleaved-cysteinyl aspartate specific proteinase (cleaved-caspase-3) were elevated while miR-137 was overexpressed, B-cell lymphoma-2 (BCL2) was concurrently reduced (Fig. 1H-J).

### Overexpression of miR-137 promotes anoikis of PC cells *in vivo*

**Figure 2:**
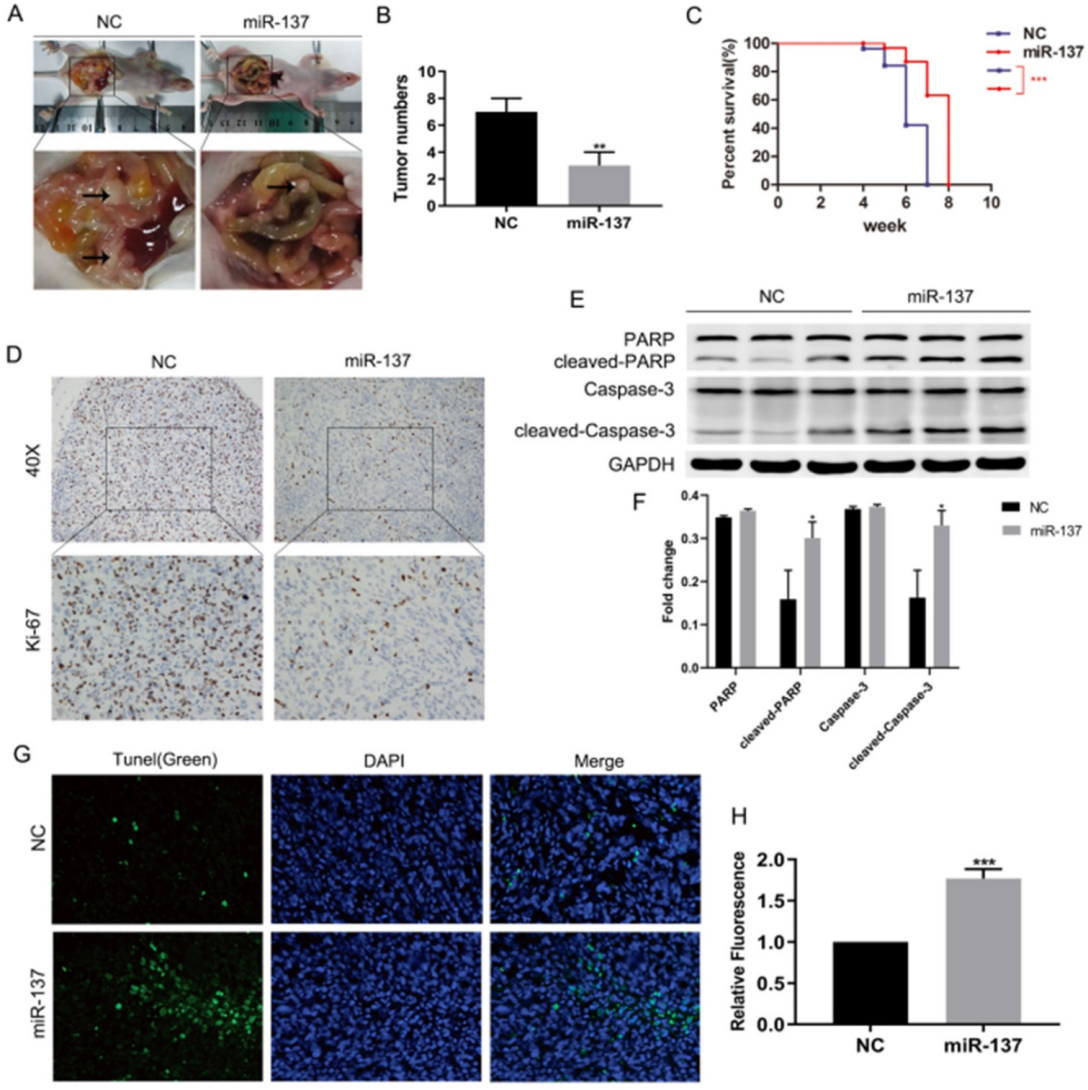
** Overexpression of miR-137 promotes anoikis of PC cells *in vivo*. A.** Representative images of the nude mice and xenografts inoculation in miR-137 and NC groups. **B.** The average tumor numbers of miR-137 and NC groups. **C.** Analysis of the survival rates of miR-137 and NC groups. **D.** Immunohistochemical images of Ki-67 expression in miR-137 and NC groups. **E-F.** The expression of the apoptosis proteins including PARP, cleaved PARP, Caspase-3 and cleaved Caspase-3 in miR-137 and NC groups. **G-H.** Tunel assays to detect the apoptosis cells of implanted tumors and captured by confocal microscopy. *P<0.05; **P<0.01; ***P<0.001.

To validate whether miR-137 could promote anoikis of PC cells *in vivo*, panc-1 cells stably expressing miR-137 (miR-137 group) and empty vector (NC group) were intraperitoneally injected into 4-week-old BALB/c nude mice, the number and size of xenograft tumors in abdominal cavity of miR-137 group were less than NC group (Fig. 2A-B). Surviving curve assays showed nude mice of miR-137 group survived longer than NC group (Fig. 2C). Compared to the NC group, immunohistochemical assays displayed ki-67 was downregulated in miR-137 group (Fig. 2D). Furthermore, western blot assays showed apoptotic protein including cleaved-PARP and cleaved-caspase-3 of miR-137 group implanted tumors expressed more (Fig. 2E-F). Meanwhile, tunel assays indicated the apoptotic rate of implanted tumors in miR-137 group was higher than NC group (Fig. 2G-H). Taken together, overexpression of miR-137 could promote anoikis of PC cells *in vivo*.

### PXN is a critical target of miR-137

**Figure 3:**
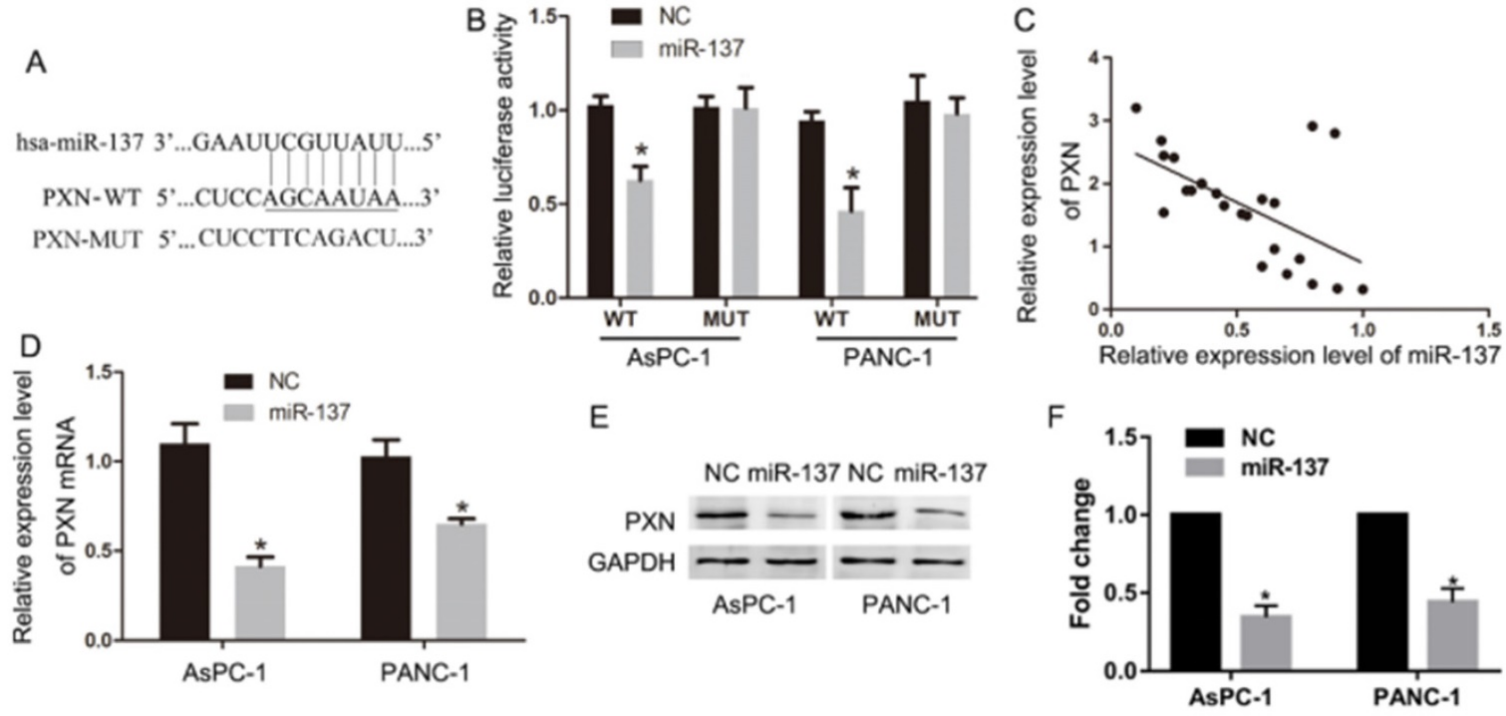
** PXN is a critical target of miR-137. A.** The potential target genes of miR-137 predicted by Target Scan. **B.** A reporter vector containing the wild-type or MUT (mutant) PXN 3′-UTR was used to transfect the indicated cells, along with the negative control or the LV-miR-137. Luciferase activity, normalized to that of renilla luciferase, was measured in three independent experiments 48 h after transfection. **C.** The correlation of PXN mRNA and miR-137 levels in 23 PC tissues. The Pearson product-moment correlation coefficient and significance levels are indicated. **D-F.** QPCR and western blot assays were performed to determine the levels of PXN expression in NC and miR-137 groups; *P<0.05.

To investigate the target genes of miR-137, we performed Target Scan to predict that miR-137 targeted the 3′-UTR of the PXN mRNA (Fig. 3A). Coincidently, luciferase assays suggested that the signal emitted by the pGL3-3′UTR-PXN luciferase reporter was less intense compared with that of the pGL3-3′UTR-PXN mut luciferase gene reporter (Fig. 3B). In addition, qPCR analysis revealed a significant (*P<*0.05, R = 0.68) inverse correlation between the expression of miR-137 and PXN in 23 samples acquired from patients with PC (Fig. 3C). Meanwhile, qPCR and western blot assays revealed that miR-137 significantly inhibited the expression of PXN mRNA and protein (Fig. 3D-F).

### PXN inhibited anoikis of PC cells

**Figure 4:**
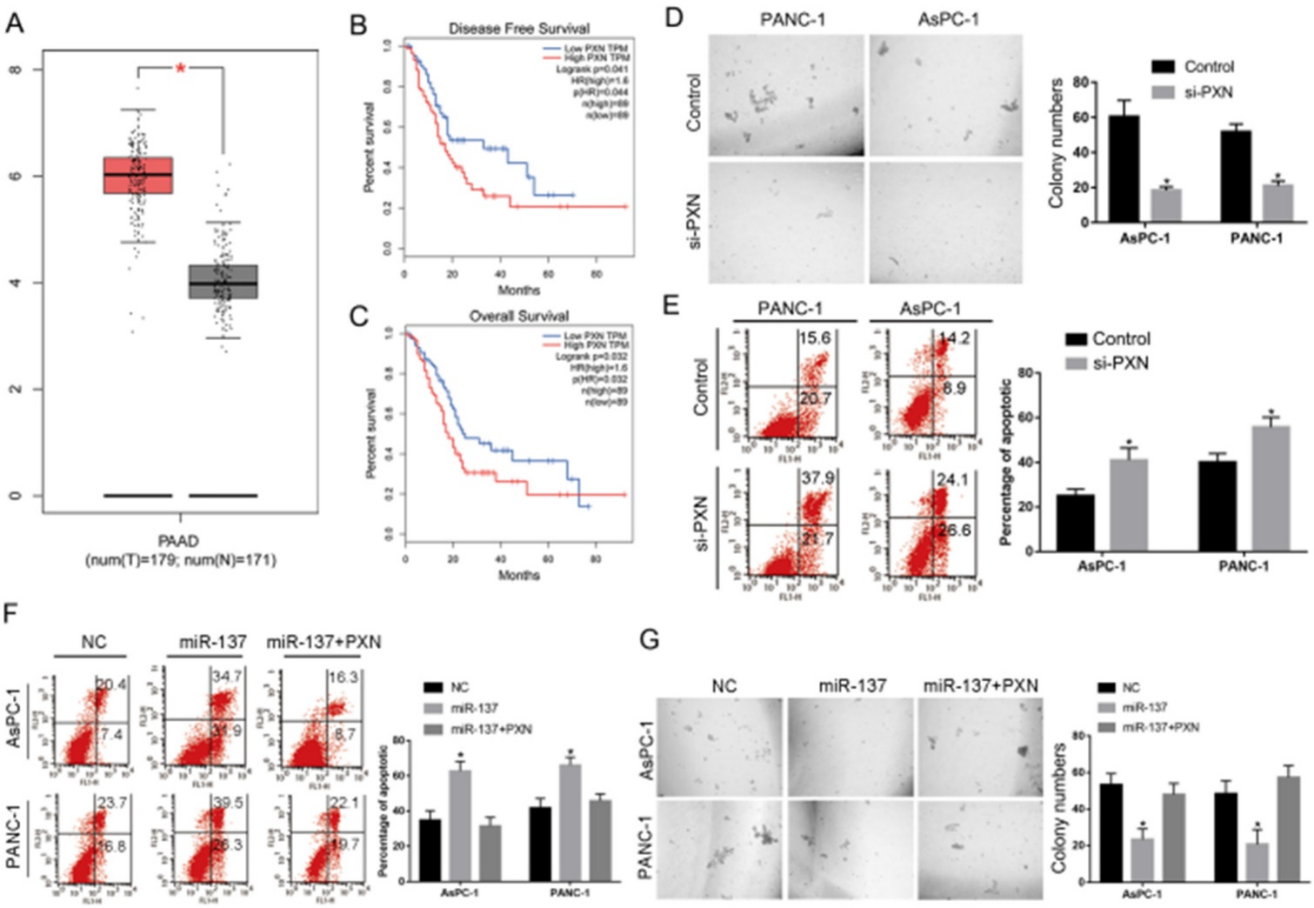
** PXN inhibited anoikis of PC cells. A.** Relative PXN expression levels assessed in PC tissues according to TCGA database. **B-C.** Kaplan-Meier analysis of disease-free survival (B) and overall survival (C) in pancreatic cancers according to TCGA database. **D.** The anoikis activities of AsPC-1 and PANC-1 cells transfected with NC or si-PXN were evaluated by suspension culture assays. **E-F.** Flow cytometry assay analysis of the apoptosis rate of the pancreatic cancer cells anoikis in the indicated groups. **G.** Suspension culture analysis the clone growth of the pancreatic cancer cells anoikis in the indicated groups; *P<0.05.

As the PXN was the function target gene of miR-137 in PC, we analyzed its expression in clinical samples containing 171 normal and 179 PC tissues from the Cancer Genome Atlas databases (TCGA), and PXN expression significantly upregulated by 1.5-fold in PC tissues (Fig. 4A). Moreover, high levels of PXN expression correlated with poor prognosis (Fig. 4B, C). To further investigate the role of PXN in anoikis of PC cells, suspension culture assays reveals that knockdown of PXN levels significantly decreased clonogenicity of the anoikis of PC cells (Fig. 4D). Flow cytometry analysis demonstrated that PXN knockdown significantly promoted the apoptosis rate in the pancreatic cancer cells anoikis (Fig. 4E). Furthermore, we investigated whether PXN is the function target of miR-137 mediated pancreatic cancer cell anoikis resistance, the rescue experiments were carried out with miR-137 mimics and PXN overexpression plasmid. Suspension culture and flow cytometry assays elucidated that miR-137 inhibited clone growth and promoted the apoptosis rate in the pancreatic cancer cells anoikis, while the PXN overexpression reversed the phenotype (Fig. 4F, G).

### The AKT signal transduction pathway plays an essential role in the PXN to induce anoikis of PC cells

**Figure 5:**
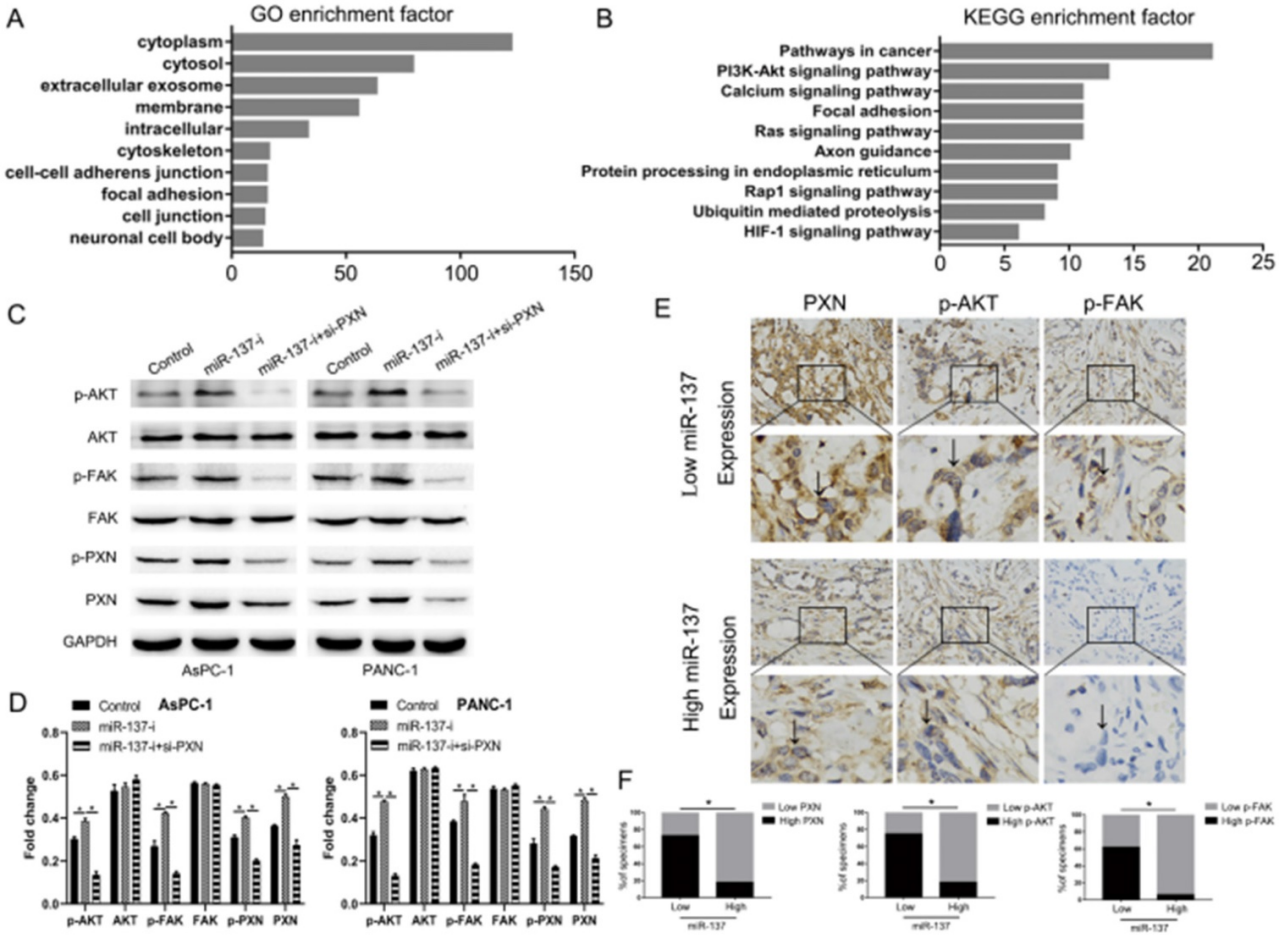
** The AKT signal transduction pathway plays an essential role in the PXN to induce anoikis of PC cells. A.** GO analysis of the TCGA PC dataset indicated the putative function of PXN. **B.** KEGG pathway analysis of the TCGA PC dataset showed the putative pathway transduced the PXN signal. **C-D.** Western blot analysis of the AKT pathways proteins expression. **E-F.** Immunohistochemical analysis of PXN, p-FAK, p-AKT expression in low or high levers miR-137 of PC tissues; *P<0.05.

The data presented above strongly suggested that miR-137 promoted the anoikis of PC cells through inhibiting the expression of PXN. We performed pathway analysis by the TCGA databases and found that PXN significantly correlated with cell adhesion function and the AKT signaling pathway (Fig. 5A, B). Downregulation of miR-137 promoted the expression of the phosphorylation of AKT (p-AKT) without altering the levels of their unphosphorylated forms, but the expression of p-AKT in miR-137-i+si-PXN group was downregulated, indicating these pathways were functional downstream of PXN (Fig. 5C, D). Strikingly, immunohistochemical assays showed that high levers of miR-137 expression of patients expressed less PXN, p-AKT and p-FAK (Fig. 5E, F). To sum up, the AKT signal transduction pathways were downstream of PXN.

## Discussion

In this study, we present several surprising findings concerning the critical role of miR-137 in metastasis progression of PC. PC is highly invasive malignancies and approximately 50% of patients with PC have metastases at diagnosis and thence the underlying mechanisms of PC metastasis are critical. Anoikis resistance is a critical contributor to tumor invasion and metastasis 17. But the role of miR-137 on anoikis is still unclear. In this context, we explore the mechanisms of miR-137 on anoikis.

Firstly, overexpression of miR-137 promoted anoikis of PC cells *in vitro* and vivo. A great amount of evidence indicates that miRNAs act as tumor suppressors or oncogenes. MicroRNA-296 targets AKT2 in PC and functions as a potential tumor suppressor 20. In contrast, microRNA-193a stimulates PC cells repopulation and metastasis through modulating TGF-β2/TGF-βRIII signaling 21. To date, the role of miR-137 on anoikis remains mysterious in PC. It is previously reported that the expression of miR-137 was significantly downregulated in certain cancer tissues including PC 22. We demonstrated that miR-137 can accelerate anoikis of PC cells. In support of this result, it has been reported that miR-10a suppressed colorectal cancer metastasis by modulating the epithelial-to-mesenchymal transition and anoikis 23 and miR-145 promoted anoikis resistance in tumor endothelial cells 24. Based on these conclusions, we proposed that miR-137 promoted anoikis of PC cells *in vitro* and vivo. Then these results we did verified such.

Secondly, miR-137 is a new regulator of PXN in PC progression. Accumulated evidence showed that miRNAs regulated PXN including microRNA-212 25, microRNA-145 26, microRNA-218 27. Moreover, miR-137 impaired the proliferative and migratory capacity of human non-small cell lung cancer cells by targeting PXN 28. In this study, we found that miR-137 promoted anoikis of PC cells by inhibiting PXN. Target Scan analysis showed that miR-137 bound to PXN mRNA. Luciferase assays supported this prediction. Then the correlation in anoikis of PC cells between miR-137 and PXN was detected. Results showed that miR-137 inhibited PXN expression. Consistently, PXN inhibited anoikis of PC cells which function was opposite to miR-137. Thirdly, miR-137 regulated AKT signal transduction pathways on anoikis of PC cells. TCGA analysis showed that AKT pathways were associated with PXN. It is reported that PXN disassembly from focal adhesions and the binding of the phosphatase PEST to PXN have been shown to play a key role in cell migration 14. To support these assumptions in PC, we did western blotting and immunohistochemistry assays. Results showed miR-137 regulated AKT signal transduction pathways on anoikis of PC cells.

In summary, results showed overexpression of miR-137 promoted anoikis of PC cells *in vitro* and vivo, and regulated AKT signal transduction pathways on anoikis of PC cells. These findings provide new insights to the underlying mechanism of metastasis.
